# Navigation accuracy and assessability of carbon fiber-reinforced PEEK instrumentation with multimodal intraoperative imaging in spinal oncology

**DOI:** 10.1038/s41598-022-20222-7

**Published:** 2022-09-22

**Authors:** Vanessa Hubertus, Lars Wessels, Anton Früh, Dimitri Tkatschenko, Irini Nulis, Georg Bohner, Vincent Prinz, Julia Onken, Marcus Czabanka, Peter Vajkoczy, Nils Hecht

**Affiliations:** 1 Department of Neurosurgery, Charité – Universitätsmedizin Berlin, Corporate Member of Freie Universität Berlin, Humboldt-Universität Zu Berlin, Berlin Institute of Health, Charitéplatz 1, 10117 Berlin, Germany; 2 Department of Neuroradiology, Charité – Universitätsmedizin Berlin, Corporate Member of Freie Universität Berlin, Humboldt-Universität Zu Berlin, Berlin Institute of Health, Berlin, Germany; 3 Department of Neurosurgery, Goethe University Frankfurt, Frankfurt am Main, Germany

**Keywords:** Bone imaging, Three-dimensional imaging, Tomography

## Abstract

Radiolucent carbon-fiber reinforced PEEK (CFRP) implants have helped improve oncological follow-up and radiation therapy. Here, we investigated the performance of 3D intraoperative imaging and navigation systems for instrumentation and precision assessment of CFRP pedicle screws across the thoraco-lumbar spine. Thirty-three patients with spinal tumors underwent navigated CFRP instrumentation with intraoperative CT (iCT), robotic cone-beam CT (rCBCT) or cone-beam CT (CBCT) imaging. Two different navigation systems were used for iCT-/rCBCT- and CBCT-based navigation. Demographic, clinical and outcome data was assessed. Four blinded observers rated image quality, assessability and accuracy of CFRP pedicle screws. Inter-observer reliability was determined with Fleiss` Kappa analysis. Between 2018 and 2021, 243 CFRP screws were implanted (iCT:93, rCBCT: 99, CBCT: 51), of which 13 were non-assessable (iCT: 1, rCBCT: 9, CBCT: 3; *p = 0.0475; iCT vs. rCBCT). Navigation accuracy was highest using iCT (74%), followed by rCBCT (69%) and CBCT (49%) (*p = 0.0064; iCT vs. CBCT and rCBCT vs. CBCT). All observers rated iCT image quality higher than rCBCT/CBCT image quality (*p < 0.01) but relevant pedicle breaches were reliably identified with substantial agreement between all observers regardless of the imaging modality. Navigation accuracy for CFRP pedicle screws was considerably lower than expected from reports on titanium implants and CT may be best for reliable assessment of CFRP materials.

## Introduction

For patients with spinal oncological disease, posterior pedicle screw fixation with or without circumferential decompression, en-bloc spondylectomy, or anterior cage reconstruction represents an accepted strategy to address tumor burden, cord compression, instability and pain^1–7^. Against this background, technology in the field of spine surgery has evolved towards the development of new implant materials and computer-assisted image guidance: Carbon fiber reinforced PEEK (CFRP) has been reported to have biomechanical screw properties comparable to standard titanium implants^8^ with distinct advantages of reduced imaging artifacts^9^, less perturbation effects^10^ and the potential to significantly improve planning, safety and quality of adjuvant radiotherapy and follow-up^10–16^. Real-time spinal navigation with intraoperative 3D imaging has improved pedicle screw accuracy^17–22^, reduced radiation exposure for the OR team^23^, and holds promise to improve outcomes^24^. Importantly, the utilization of state-of-the-art intraoperative 3D imaging also permits immediate implant control and direct revision, if needed. Three of the most widely used intraoperative imaging solutions are the mobile AIRO intraoperative CT (iCT)^25^, the permanently installed Zeego robotic cone-beam CT (rCBCT)^26^, and the mobile O-arm cone-beam CT (CBCT)^27,28^. So far, little is known about the performance of using intraoperative 3D imaging in the context of CFRP pedicle screw implantation. This is highly relevant, however, because performance may differ compared to titanium screws due to the radiolucency and specific surgical nuances are required for CFRP screw insertion^16,29^. Therefore, in the present study we describe the performance of iCT, rCBCT- and CBCT-based spinal navigation for CFRP screw implantation in patients suffering oncologic spinal disease.

## Materials and methods

All methods were performed in accordance with the relevant guidelines and regulations. This single-center retrospective cohort study was conducted according to the World Medical Association Declaration of Helsinki in compliance with Health Insurance Portability and Accountability Act regulations and approved by the local ethics committee of the Charité—Universitätsmedizin Berlin (EA4/046/16 and EA4/063/20). Between January 2018 and March 2021, 33 patients with oncological spinal pathologies underwent implantation of 243 CFRP pedicle screws using iCT-, rCBCT- or CBCT-based real-time spinal navigation. The inclusion criteria for this retrospective analysis were spinal metastases or primary spinal tumors that were treated with navigated instrumentation using CFRP pedicle screw implants with or without combined corpectomy at the level of the thoracic or lumbar spine. The choice of intraoperative imaging modality was based on logistical considerations and availability. Importantly, specific anatomical or pathological considerations like the anatomic region or type of tumor did not affect the choice of imaging. The decision to use CFRP implants was based on availability and the presence of oncological spinal disease requiring posterior pedicle screw fixation with high likelihood of requiring continuous oncological imaging follow-up and/or adjuvant irradiation therapy planning. Clinical, demographic and tumor data according to the NOMS framework^30^, as well as surgical data, navigation accuracy and screw assessability for each imaging modality were retrospectively analyzed. Informed consent was waived by the ethics committee of the Charité—Universitätsmedizin Berlin according to EA4/046/16 and EA4/063/20 due to the retrospective nature of the study.

### Intraoperative 3D imaging and spinal navigation

For iCT- and rCBCT-based spinal navigation, the AIRO iCT (Brainlab AG, Munich, Germany) and robotic 3D Artis Zeego II digital fluoroscopy C-arm system (Siemens Healthcare, Forchheim, Germany) were used as previously described^25,31,32^. For CBCT-based spinal navigation, the mobile O-arm system (Medtronic plc, Dublin, Ireland) was used. For navigated pedicle screw implantation, an image-guidance system and infrared tracking camera with automatic patient/image co-registration was used (iCT and rCBCT: Brainlab Curve and Brainlab Spinal Navigation Software Version 3.0, Brainlab AG, Munich, Germany; CBCT: Stealth Station S7 Navigation System, Medtronic plc, Dublin, Ireland)^28^.

### CFRP pedicle screw implantation

**Figure 1:**
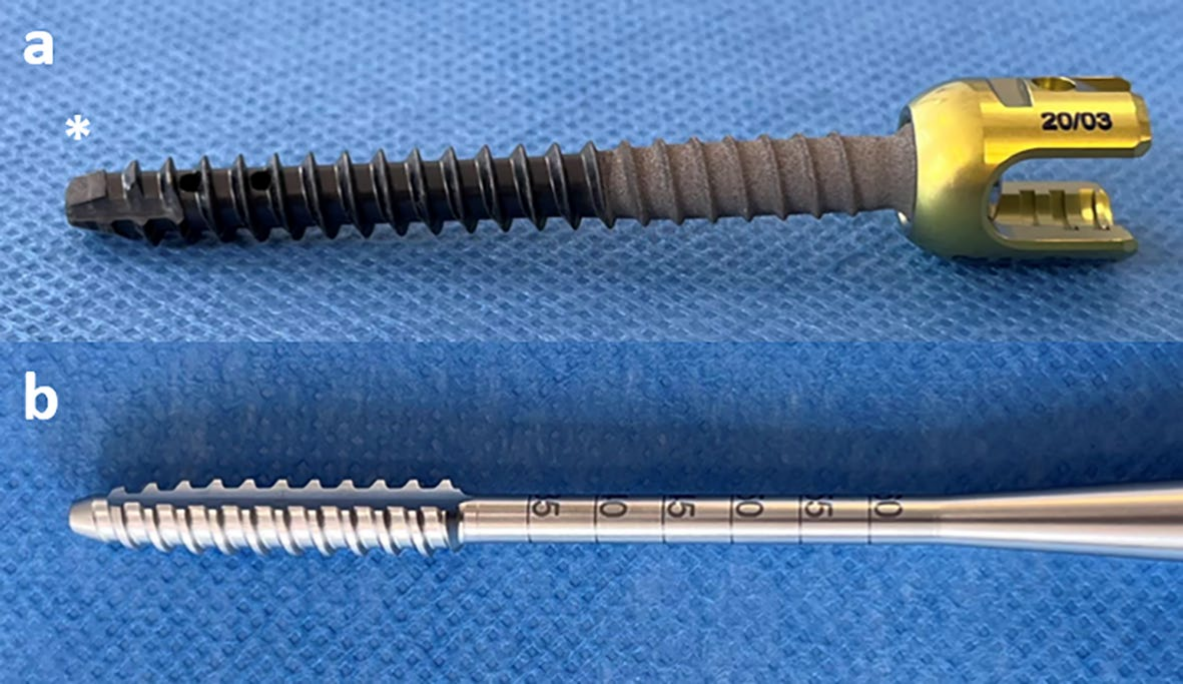
(**a**) Illustration of a 6.5 mm CFRP pedicle screw and (**b**) the corresponding screw tap showing the surface structure and design of the screw head (asterisk) and tap threads (BlackArmor, Icotec, Altstätten, Switzerland).

Surgery was performed on mobile, radiolucent, carbon-fiber examination tables (TRUMPF Carbon FloatLine or TRUMPF Carbon X-TRA, TRUMPF Medizin Systeme GmbH & Co. KG, Saalfeld, Germany). For all rCBCT procedures, the patients’ head was fixed in a radiolucent carbon fiber 3-pin head clamp (TRUMPF X-RAY, TRUMPF Medizin Systeme GmbH & Co. KG, Saalfeld, Germany). Surgical exposure was gained through a standard midline approach and a navigation reference clamp (Brainlab AG, Munich, Germany or Medtronic plc, Dublin, Ireland) was attached to a spinous process. The screw entry point and trajectory were identified with a navigated drill-guide (iCT and rCBCT: Brainlab AG, Munich; CBCT: Medtronic plc, Dublin, Ireland). A battery-powered drill (Stryker Cordless Driver, Stryker, Kalamazoo, Michigan, USA) with a 2.6 mm drill bit was used to drill a pilot hole down to a desired depth. Next, a guide-wire was inserted, the pedicle was tapped, and a cannulated, CRFP pedicle screw with a diameter between 5.5 and 7.5 mm (BlackArmor, Icotec, Altstätten, Switzerland) was inserted (Fig. 1). Screw positioning was directly assessed by a second, navigated iCT, rCBCT or CBCT scan with the chance of immediate repositioning, followed by a final iCT, rCBCT or CBCT scan, based on which the overall navigation accuracy was determined^28^.

### Blinded, inter-observer analysis of screw accuracy and assessability

Definite screw accuracy rates and information on general screw assessability were based on the assessment obtained from the most experienced *Expert* observer, according to the 2 mm-increment method initially described by Gertzbein and Robbins^33^ and modified according to Rampersaud^34^. Categories A (completely within the pedicle) and B (< 2 mm pedicle breach) were classified as *accurate* and categories C (2–4 mm pedicle breach) and D (> 4 mm pedicle breach) as *inaccurate* placement.

For the analysis of inter-observer reliability, anonymized, intraoperative imaging data sets of all patients were generated from the hospital PACS system. In addition to anonymization, patient data was blinded towards the used intraoperative imaging modality (iCT, rCBCT or CBCT). The anonymized and blinded image data sets were then distributed to four independent observers: two observers on expert-level (specialized spine surgeons), one senior PGY6 resident (6th year of neurosurgical residency training), and one PGY1 junior resident (1st year of neurosurgical residency training). Image assessment was performed with a dedicated DICOM viewer (RadiAnt DICOM viewer Version 2021.1, Medixant, Poznan, Poland). For every observer and each patient, the perceived image quality (excellent: +++, good: ++, fair: +), general screw assessability (yes/no), perceived pedicle screw accuracy (grading according to modified Rampersaud A-D), as well as the time required for accuracy assessment beginning from the 3D image reconstruction until the completion of the assessment (minutes) were determined. Screw accuracy and assessability analysis of each observer was compared for its consistency with calculation of Fleiss’ Kappa inter-observer agreement.

### Data management and statistical analysis

For data management and blinded analysis, REDCap (Research Electronic Data Capture consortium, Vanderbilt University) was used. Anonymized and blinded DICOM datasets were stored on encrypted portable devices. For statistical analysis, SPSS Version 25 (IBM, Armonk, NY, USA) and GraphPad Prism Version 9 (GraphPad Software, San Diego, CA, USA) were used. All tests were two-sided. Statistical significance was set at p < 0.05 and tested by one-way ANOVA with Bonferroni correction for multiple comparisons or Kruskal–Wallis test with Dunn’s correction for multiple comparisons, depending on normal distribution according to the Shapiro–Wilk test. Inter-observer reliability was tested with Fleiss’ Kappa analysis, with interpretation of reliability by Landis & Koch (< 0 less than chance, 0.01–0.2 slight, 0.21–0.4 fair, 0.41–0.6 moderate, 0.61–0.8 substantial, 0.81–0.99 almost perfect agreement).

### Ethics approval

The study was approved by the ethics committee of the Charité-Universitätsmedizin Berlin, Germany (EA4/046/16 and EA4/063/20).

### Consent to participate

 Waived due to the retrospective nature of the study.

## Results

**Figure 2:**
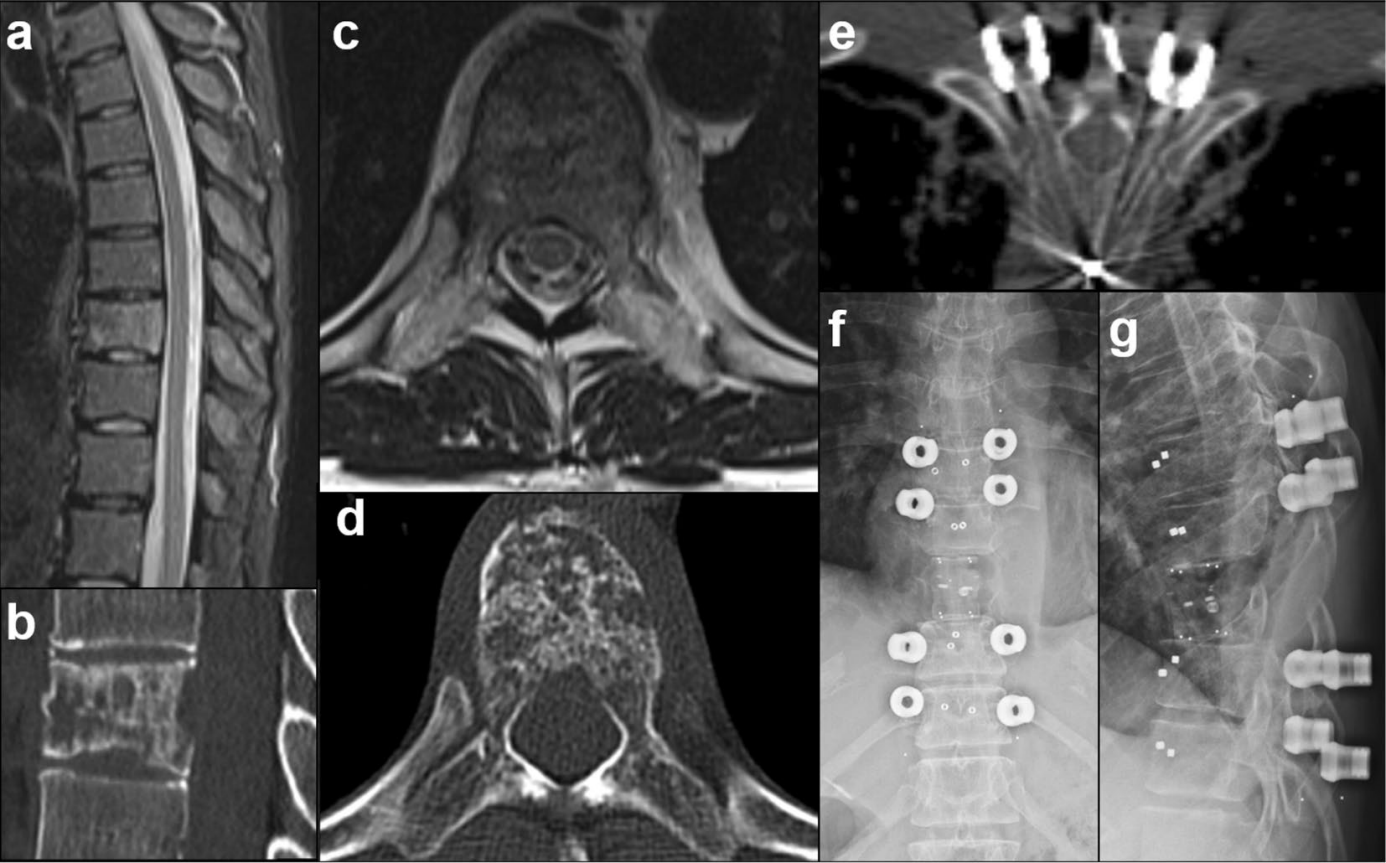
Case example of a 48-year-old female suffering from metastatic breast cancer with a singular metastasis at Th8 (SINS 7, preop. images **a–d**). The patient suffered from isolated thoracic back pain without neurological deficits and a KPS of 90%. She was treated by en-bloc spondylectomy of Th8 with CFRP vertebral body replacement and iCT-based navigated posterior instrumentation from Th6 to Th10 using CFRP pedicle screws (intraop. imaging **e** and postop. X-ray **f + g**). Adjuvant stereotactic radiosurgery was performed using CyberKnife.

Detailed demographic, clinical, tumor and outcome data are displayed in Table 1. Between January 2018 and March 2021, 243 navigated CFRP pedicle screws were implanted and assessed with iCT (93), rCBCT (99), or CBCT (51) imaging in 33 patients. Surgical data according to the intraoperatively used imaging modality are presented in Table 2. Baseline characteristics regarding the median instrumentation length, surgical technique, duration of surgery and the number of scan procedures did not differ. Figure 2 illustrates the case of a 48-year-old female suffering from metastatic breast cancer and a singular metastasis at Th8, which was treated with en-bloc spondylectomy of Th8 and iCT-based navigated posterior instrumentation of Th6-10 using CFRP pedicle screws, cage and rod.

**Table 1:** Demographic, clinical and outcome data.

	iCT	rCBCT	CBCT	p value
No. of patients	13	13	7	n/a
Age (years)	59 (18–76)	61 (34–85)	70 (60–79)	0.1227
Sex, n (%)	F 8 (62%) M 5 (38%)	F 3 (23%) M 10 (77%)	F 4 (57%) M 3 (43%)	0.1189
Primary tumor, n (%)	Lung 1 Prostate 3 Kidney 1 Breast 4 Ovary 1 Lymphoma 1 Melanoma 1 Osteoblastoma 1	Lung 5 Prostate 1 Kidney 2 Gastrointestinal 2 Myeloma 2 CUP 1	Lung 3 Prostate 1 Urothelium 1 Lymphoma 1 Chordoma 1	n/a
Spinal segments, n (%)	Thoracic 13 (100%)	Thoracic 12 (92%) Lumbar 1 (8%)	Thoracic 4 (57%) Lumbar 3 (43%)	*0.0134 iCT vs. CBCT
Median SINS at index level	8 (4–13)	7 (5–11)	9 (4–10)	0.5576
Median BMI	24 (19–30)	23 (18–29)	26 (18–30)	0.5021
Median ASA	3 (1–3)	3 (1–4)	3 (3–4)	0.2430
Neurological deficits, n (%)	4 (30%) Ataxia 1 Incomplete paralysis 3	5 (38%) Ataxia 1 Incomplete paralysis 4	2 (29%) Incomplete paralysis 1 Complete paralysis 1	0.8866
**Complications and outcome data**				
Surgical complications, n (%)	4 (30%) SSI 4	2 (15%) SSI 2	2 (29%) SSI 1 Cage dislocation 1	0.6376
Reoperation rate, n (%)	4 (30%) SSI 3 Tumor recurrence 1	3 (23%) SSI 2 Tumor recurrence 1	2 (29%) SSI 1 Cage revision 1	0.9069
Hospitalization (days)	9 (5–30)	12 (7–29)	15 (6–30)	0.2966
Adjuvant irradiation therapy, n (%)	7 (54%) CBRT 6 SRS 1	8 (62%) CBRT 7 SRS 1	6 (86%) CBRT 6	0.3723
Median days to irradiation	18 (14–31)	27 (19–53)	26 (11–43)	0.2722

Values are given in total number with percentages or as median with total range, as appropriate.

*ASA* American Society for Anesthesiology, *BMI* Body Mass Index, *CBRT* Conventional Beam Radiation Therapy, *CUP* Cancer of unknown primary, *SINS* Spinal Instability Neoplastic Score, *SRS* Stereotactic radiosurgery, *SSI* Surgical Site Infections.

**Table 2:** Surgical data depending on the intraoperative imaging modality.

	iCT	rCBCT	CBCT	p-value
Total number of surgeries	13 (39%)	13 (39%)	7 (21%)	n/a
Total number of navigated screws	93	99	51	n/a
Total number of imaged screws	93	99	51	n/a
Total number of assessable screws, n (%)	92 (99%)	90 (91%)	48 (94%)	*0.0475 (iCT vs. rCBCT)
Median number of navigated screws per patient	8 (4–12)	8 (4–10)	8 (4–10)	0.8360
Median number of instrumented segments per patient	5 (3–7)	5 (3–7)	5 (3–6)	0.8352
Decompression + Instrumentation, n (%) Decompression + Instrumentation + Corpectomy, n (%)	8 (62%) 5 (38%)	10 (77%) 3 (23%)	4 (57%) 3 (43%)	0.5996
Median duration of surgery (min)	248 (110–387)	202 (105–350)	193 (78–487)	0.7312
Median number of intraoperative scans per patient	2 (2–4)	2 (2–4)	2 (2–3)	0.6983

Values are given in total number with percentage or as median with total range, as appropriate.

*iCT* Intraoperative CT, *rCBCT* robotic cone beam CT, *CBCT* cone beam CT, *n/a* not applicable.

### Intraoperative CFRP pedicle screw assessability and accuracy rates

**Figure 3:**
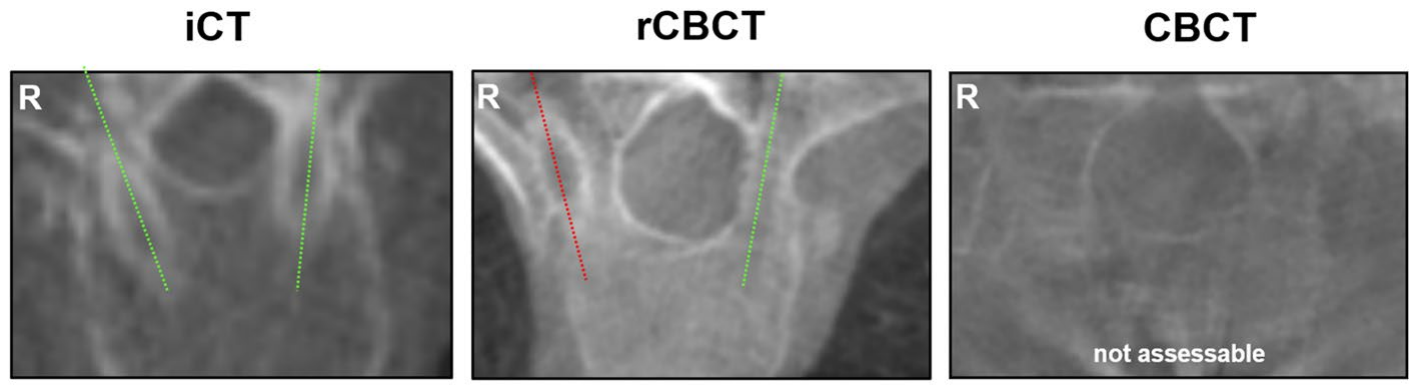
Examples of image quality and screw assessability using iCT, rCBCT and CBCT imaging in the thoracic spine. The green lines illustrate correctly placed screws (modified Rampersaud A + B) and the red line shows a misplaced screw (modified Rampersaud D). An example of non-assessable screw accuracy with CBCT imaging is shown in the right panel.

The number of generally assessable screws differed significantly between iCT (99%), rCBCT (91%) and CBCT (94%; *p = 0.0472; iCT vs. rCBCT). Six out of 93 (7%) iCT screws underwent navigated revision, in contrast to zero revised screws in the rCBCT and CBCT groups (Table 3). Examples of screw assessability are shown in Fig. 3. Similarly, screw accuracy differed significantly with the highest accuracy (category A + B) in iCT and the lowest accuracy in CBCT imaging (iCT: 74%, rCBCT: 69%, CBCT: 49%; *p = 0.0064; iCT vs. CBCT and rCBCT vs. CBCT). In breached pedicles (category B, C or D) the median pedicle isthmus diameter was lower than in pedicles without breaches (category A). Likewise, the ratio of the pedicle isthmus/screw diameter was lower in cases of breached pedicles than in cases without breaches, indicating an unfavorable isthmus-to-screw ratio. More specifically, 28 of 243 (12%) pedicle screws were larger than the corresponding pedicle isthmus by a median diameter of 0.3 mm (range 0.1–1.1 mm), mainly at the upper- to mid-thoracic level (Th2-Th9) and only twice at the upper lumbar level (L1-2). However, the overall accuracy did not substantially differ, even after screws with a diameter larger than the pedicle isthmus were excluded from the analysis (iCT: 74%, rCBCT: 73%, CBCT: 46%; *p = 0.0056; iCT vs. CBCT and rCBCT vs. CBCT). An additional, region-specific analysis based on the SIN score (Thoracic = Th1-10, Thoraco-Lumbar Junction (TLJ) = Th11-L1, Lumbar = L2-5) yielded a notably poorer screw accuracy in the Th region (iCT: 68%, rCBCT: 68%; CBCT: 25%), compared to the TLJ (iCT: 92%, rCBCT: 80%; CBCT: 63%) or L (iCT: 100%, rCBCT: 100%, CBCT: 88%) area. For iCT and rCBCT, no regional difference was noted (iCT: p = 0.0751, rCBCT: p = 0.3816). For CBCT, however, a region-specific accuracy difference was detected (CBCT: *p = 0.0202) and significant between thoracic (Th) and lumbar (L) regions (CBCT for Th vs. L: *p = 0.0176) (Table 3).

**Table 3:** CFRP screw accuracy and assessability depending on imaging modality.

	iCT	rCBCT	CBCT	p-value
Total no. of navigated screws	93	99	51	n/a
Not assessable, n (%)	1 (1%, T8)	9 (9%, T 7–12)	3 (6%, T11–12)	*0.0472 (iCT vs. rCBCT)
Screws intraoperatively corrected, n (%)	6 (7%)	0	0	0.0853
Screw accuracy (A + B), n (%)	69 (74%)	68 (69%)	25 (49%)	**0.0064 (rCBCT vs. CBCT and iCT vs. CBCT)
Pedicle breach 2–4 mm (C), n (%)	14 (15%)	19 (19%)	13 (25%)	0.3395
Pedicle breach > 4 mm (D), n (%)	9 (10%)	3 (3%)	10 (20%)	**0.0042 (rCBCT vs. CBCT)
Median pedicle isthmus diameter of instrumented pedicles without breach (A, mm)	6.5 (5.5–13)	6.5 (5.5–15)	9 (6–12)	*0.0407 (rCBCT vs. CBCT)
Median pedicle isthmus diameter of instrumented pedicles with breached screws (B–D, mm)	5.7 (3.9–8.8)	5.8 (3.4–10)	6.1 (4.5–11)	0.4982
Median isthmus/screw diameter–ratio in screws without breach (A)	1.2	1.2	1.4	0.1180
Median isthmus/screw diameter–ratio in breached screws (B–D)	1.04	1.1	1.1	0.9360
Number of pedicle screws larger than pedicle isthmus, n (%)	5 (5%)	13 (13%)	10 (19%)	*0.0353 (iCT vs. CBCT)
Screw accuracy (A + B) without screws larger than the pedicle isthmus, n (%)	67/91 (74%) Th: 39/57 (68%) TLJ: 24/26 (92%) L: 4/4 (100%) (n.s.)	56/77 (73%) Th: 41/60 (68%) TLJ: 8/10 (80%) L: 6/6 (100%) (n.s.)	17/37 (46%) Th: 5/20 (25%) TLJ: 5/8 (63%) L: 7/8 (88%) (*p = 0.0202)	**0.0056 (iCT vs. CBCT, rCBCT vs. CBCT)
Median time for accuracy assessment (seconds)	120 (27–540)	120 (17–720)	180 (24–1080)	0.5551

Values are given in total number with percentage or as median with total range, as appropriate.

*iCT* Intraoperative CT, *rCBCT* robotic cone beam CT, *CBCT* cone beam CT, *n.s.* not statistically significant. Based on SIN score: L = lumbar (L2-5), Th = thoracic (Th1-10), TLJ = thoraco-lumbar junction (Th11-L1).

### Inter-observer agreement of screw accuracy analysis

**Figure 4:**
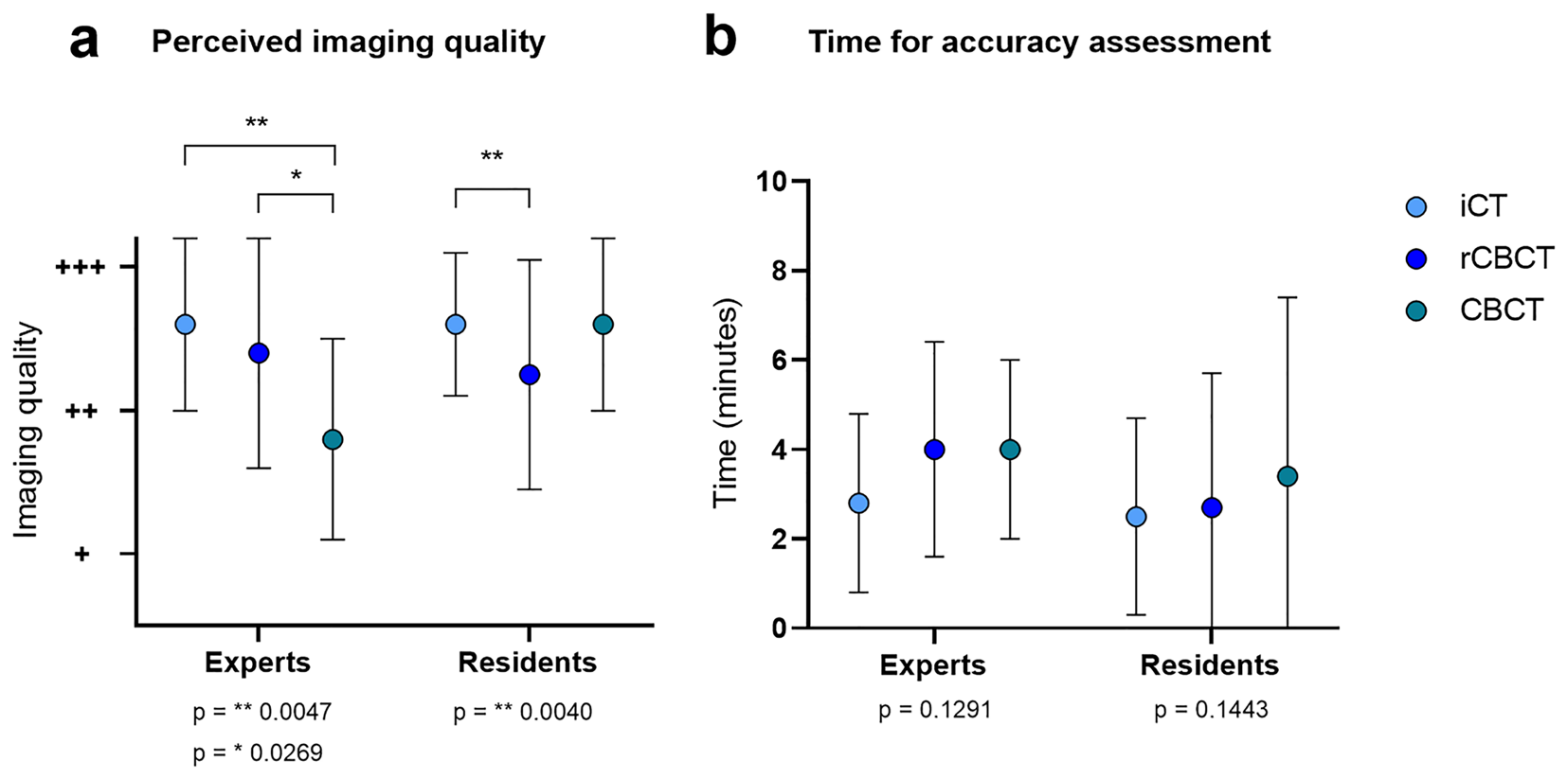
(**a**) Perceived imaging quality of the different observers (Experts and Residents), with perceived imaging quality classified as excellent (+++), good (++) and fair (+). (**b**) Mean time (minutes) required for screw accuracy assessment by the different observers (Experts and Residents).

**Figure 5:**
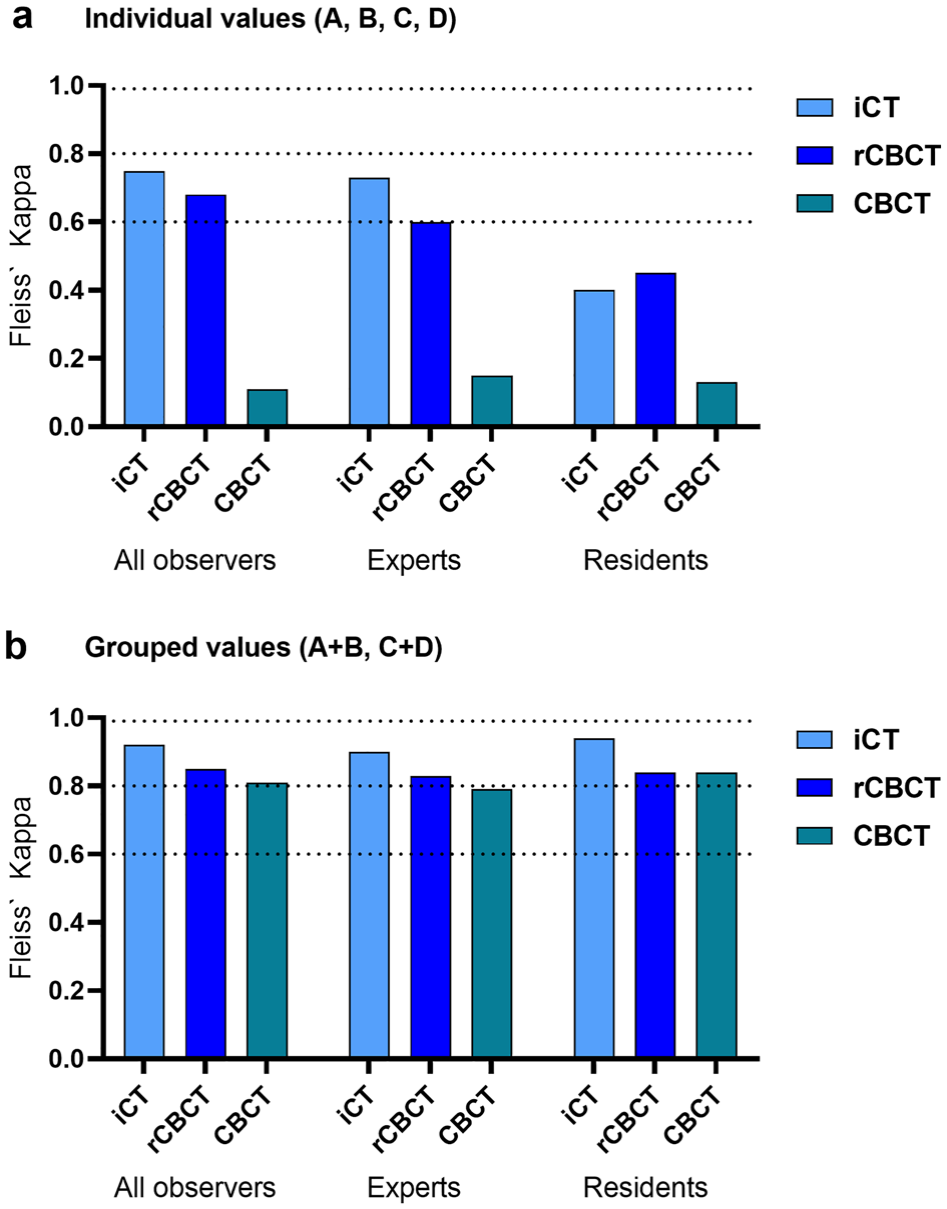
Interobserver reliability of screw accuracy depending on intraoperative imaging modality via Fleiss’ Kappa analysis. (**a**) shows the interobserver reliability of the individual accuracy analysis comparing modified Rampersaud A, B, C and D of All observers, Experts, and Residents depending on the intraoperative imaging modality. (**b**) shows the interobserver reliability of the accuracy analysis grouped according to non-relevant (modified Rampersaud A + B) and relevant (modified Rampersaud C + D) pedicle breaches. The dashed lines illustrate the interobserver reliability of substantial (> 0.6), almost perfect (> 0.8), and perfect (> 0.99) agreement according to the interpretation of reliability by Landis & Koch (< 0 less than chance, 0.01–0.2 slight, 0.21–0.4 fair, 0.41–0.6 moderate, 0.61–0.8 substantial, 0.81–0.99 almost perfect agreement).

To take the more difficult radiographic assessability of radiolucent CFRP screws into account, we then compared the perceived imaging quality and the time required for screw assessment among two different groups of observers (*Experts* and *Residents)* and found that both *Experts* and *Residents* rated the image quality of iCT higher compared to CBCT technology (*Experts:* iCT vs. CBCT **p = 0.0047 and rCBCT vs. CBCT *p = 0.027; *Residents:* iCT vs. rCBCT **p = 0.004) but this did not relevantly affect the time required for screw accuracy analysis (Fig. 4). To judge whether the interpretation of CFRP screw placement accuracy was affected by the observers’ experience, we determined the inter-observer agreement across all screw placement categories (A, B, C and D) and found that agreement was higher between *Experts* than between *Residents* and lowest for CBCT imaging, regardless of the observers’ experience (Fig. 5a). Next, we grouped screw placement categories into categories that we considered most likely to be clinically relevant (categories A + B vs. C + D) and found that both *Experts* and *Residents* now reached substantial to almost perfect agreement for each of the 3 imaging modalities (Fig. 5b).

## Discussion

The general feasibility of using radiolucent CFRP pedicle screws in spinal oncology has previously been demonstrated^8,12,16^ but little is known about the performance of state-of-the art intraoperative 3D imaging for spinal navigation and implant assessment of CFRP pedicle screws. This is important, however, because spinal navigation and robotics is gaining increasing attention and screw trajectories of CFRP implants within the pedicle are more difficult to identify. Against this background, we compared the performance of intraoperative CT, rCBCT and CBCT imaging used within the same surgical environment for standardized, navigated spinal instrumentation and implant assessment of radiolucent CFRP pedicle screws across the thoraco-lumbar spine. A main finding in our study was that perceived image quality and implant assessability differed markedly between iCT and rCBCT/CBCT technology and even despite multiplanar 3D reconstructions. The fact that perceived image quality of iCT imaging was rated higher than that of rCBCT/CBCT mirrors recent experience regarding radiographic visualization^25,28,35^. Further, direct CFRP implant assessment appeared somewhat limited using rCBCT and CBCT, which is likely explained by the fact that image quality of rCBCT / CBCT technology generally remains more susceptible to artifacts due to the larger X-ray beam and semi-circular beam rotation of CBCT technology that generates a larger amount of scatter radiation compared to fan-beam CT imaging^36^. This image quality/screw assessability difference could also explain that only screws in the iCT group underwent intraoperative revision, because the lower perceived CBCT image quality may have hampered reliable breach detection. Nevertheless, we consider the < 10% rate of non-assessable CFRP screws acceptable, in view of our previous experience with first generation CBCT technology that yielded 14% non-assessable screws using titanium implants^32^. Nevertheless, the surgeon should be prepared that not each individual CFRP screw may be reliably assessed using CBCT technology, particularly in the osteopenic spine.

The low accuracy of 49% (CBCT) to 74% (iCT) that we experienced with CFRP pedicle screws was unexpected, considering that previous studies have consistently demonstrated navigation accuracy rates above 90% for titanium implants using each of the three investigated imaging modalities^27,31,32,35^ and that all procedures were performed according to the same workflow and by a group of similarly trained spine surgeons with experience in spinal navigation. Also, the accuracy difference between groups was at least partially due to the variable image quality between iCT and rCBCT / CBCT, which may have affected navigation accuracy particularly in cases of difficult radiographic visualization, such as osteolytic bone, obesity or in the upper to mid thoracic spine. The fact that we did not perform routine postoperative CT imaging in each case to validate our findings remains a clear limitation. However, the following aspects could have affected accuracy independent from using image guidance: First, the generally low accuracy in all groups could be an indirect effect of the improved radiographic visualization of radiolucent CFRP screws, which in cases of sufficient image quality for screw assessment (> 90% in all groups) permitted highly sensitive and specific identification and categorization of even the slightest pedicle breaches. In particular, radiolucent CFRP implants offered a completely distinct and sharp view of the pedicle isthmus outline and permitted highly precise measurement and breach categorization according to the perception of the most experienced *Expert* observer, who graded the baseline categorization. Since the majority of all misplacements were noted within the range of 2–4 mm (category C), such highly sensitive breach detection and categorization might have unmasked category B (or C) CFRP screws that would otherwise have been graded as category A (or B) using titanium implants. Second, implantation of CFRP pedicle screws requires knowledge of certain technical nuances for successful implantation, such as meticulous selection of the screw entry point in order to ensure precise alignment, given the inability to bend the pre-shaped carbon fiber rods that we used^16^. Third, in oncologic spinal disease we typically aim to implant the largest possible screw diameter just below the pedicle isthmus diameter to ensure maximum pullout resistance and limit the risk of a pedicle breach. In the present cohort, the median pedicle isthmus diameter of breached pedicles ranged between 5.7 and 6.1 mm for all imaging modalities (Table 3). This was merely 0.2–0.6 mm larger than the smallest available CFRP screw diameter (5.5 mm) and practically eliminated the room for error regarding correct screw placement, particularly in the thoracic spine. Here, manufacturing of smaller implant diameters could help to reduce the rate of breaches. However, after exclusion of screws larger than the pedicle isthmus, the noted accuracy was comparable to our initial analysis. This on the other hand suggests that the low accuracy rates were most likely not primarily due to an unfavorable screw/isthmus diameter ratio. Possibly, this effect could be better explained by the results from our region-specific analysis, which demonstrated that the high inaccuracy rate was mainly localized in the thoracic region and clearly underlines that thoracic accuracy requirements remain among the highest^37^. Further, the perceived lower CBCT image quality determined by blinded assessment may have limited the navigation accuracy particularly in that group. Of course, these results need to be interpreted with caution given the low sample size but taken together, these factors could at least partially account for the poor accuracy that we noted and warrant a future direct comparison to titanium implants using the same navigation technology in all cases. Importantly, in all cases the noted pedicle breaches did not cause any clinical harm, such as neurovascular injury or biomechanical failure with the need for secondary screw revision surgery (Table 1).

Inadequate training is one of the main reasons cited when spine surgeons refrain from adapting image-guidance^38^ and we believe that continuous exposure is required to maintain a high level of performance and ensure training of the entire surgical team, including spine surgeons and residents with different levels of experience. The radiolucency of CFRP implant materials present a challenge regarding simple, fast and reliable assessment of pedicle breaches. Therefore, we determined the inter-observer reliability between resident and expert observers as well as the perceived image quality and required time for accuracy assessment of CFRP pedicle breaches based on iCT, rCBCT and CBCT imaging. The finding that residents generally rated screw placement accuracy with less agreement than experts and that agreement was lowest for CBCT imaging seems intuitive, given the likely greater experience of fully trained spine surgeons in judging pedicle screw accuracy compared to residents in training and the generally lower perceived image quality of CBCT compared to iCT. On the other hand, the fact that residents had substantial to almost perfect agreement when grading breaches considered most likely to be clinically relevant (categories C and D) and that this high level of agreement was reached for each imaging modality, including CBCT, nicely shows that each of the imaging modalities is feasible for reliable detection of clinically relevant breaches, regardless of the observers’ level of experience and despite differences in image quality.

### Limitations

The retrospective design of our study and small sample size bears well known limitations and lacks systematic outcome assessment, including pain and Quality of Life scores and follow-up regarding implant durability. Another major limitation is that the individual effective radiation (organ) doses applied by each modality were not directly measured, so that individual radiation exposure was not comparable, because radiation dosage in fan-beam CT (iCT) and cone-beam CT (rCBCT/CBCT) are recorded in different dosage units (iCT: dose-length-product [mGy/cm]; rCBCT/CBCT: dose-area-product [mGycm^2^]), which prohibits a direct comparison of system-documented radiation dosage.

## Conclusion

Accuracy of navigated CFRP pedicle screws was considerably lower than expected from previous navigation experience with titanium implants and CFRP screw assessability as well as the interrater-reliability of CFRP screw assessment were affected by the type of imaging modality and the experience of the observer. Overall, iCT yielded the highest navigation precision, best perceived image quality and permitted robust CFRP implant assessment independent from the users’ experience. The present study shows that the choice of intraoperative 3D imaging for navigated CFRP pedicle screw instrumentation has significant impact on standard procedures in the field of computer assisted spine surgery.

## Data Availability

Supporting data is available from the corresponding author upon reasonable request.

## References

[1] Patchell RA (2005). Direct decompressive surgical resection in the treatment of spinal cord compression caused by metastatic cancer: A randomised trial. Lancet.

[2] Hubertus V (2021). Surgical management of spinal metastases involving the cervicothoracic junction: Results of a multicenter, European observational study. Neurosurg. Focus.

[3] Bilsky MH, Laufer I, Burch S (2009). Shifting paradigms in the treatment of metastatic spine disease. Spine.

[4] Sciubba DM (2010). Diagnosis and management of metastatic spine disease. J. Neurosurg. Spine.

[5] Cloyd JM, Acosta FL, Polley M-Y, Ames CP (2010). En bloc resection for primary and metastatic tumors of the spine. Neurosurgery.

[6] Barzilai O, Fisher CG, Bilsky MH (2018). State of the art treatment of spinal metastatic disease. Neurosurgery.

[7] Fehlings MG, Ahuja CS, Mroz T, Hsu W, Harrop J (2017). Future advances in spine surgery: The AOSpine North America perspective. Neurosurgery.

[8] Lindtner RA, Schmid R, Nydegger T, Konschake M, Schmoelz W (2018). Pedicle screw anchorage of carbon fiber-reinforced PEEK screws under cyclic loading. Eur. Spine J..

[9] Fleege C (2020). Carbon fiber-reinforced pedicle screws reduce artifacts in magnetic resonance imaging of patients with lumbar spondylodesis. Sci. Rep..

[10] Nevelsky A, Borzov E, Daniel S, Bar-Deroma R (2017). Perturbation effects of the carbon fiber-PEEK screws on radiotherapy dose distribution. J. Appl. Clin. Med. Phys..

[11] Boriani S (2018). Carbon-fiber-reinforced PEEK fixation system in the treatment of spine tumors: A preliminary report. Eur. Spine J..

[12] Ringel F (2017). Radiolucent carbon fiber-reinforced pedicle screws for treatment of spinal tumors: Advantages for radiation planning and follow-up imaging. World Neurosurg..

[13] Müller BS (2020). The dosimetric impact of stabilizing spinal implants in radiotherapy treatment planning with protons and photons: Standard titanium alloy vs. radiolucent carbon-fiber-reinforced PEEK systems. J. Appl. Clin. Med. Phys..

[14] Laux CJ, Hodel SM, Farshad M, Müller DA (2018). Carbon fibre/polyether ether ketone (CF/PEEK) implants in orthopaedic oncology. World J. Surg. Oncol..

[15] Krätzig T (2020). Carbon fiber–reinforced PEEK versus titanium implants: An in vitro comparison of susceptibility artifacts in CT and MR imaging. Neurosurg. Rev..

[16] Neal MT (2021). Carbon fiber–reinforced PEEK instrumentation in the spinal oncology population: A retrospective series demonstrating technique, feasibility, and clinical outcomes. Neurosurg. Focus.

[17] Ryang, Y.-M., Obermüller, T. & Friedrich, B. Learning curve of 3D-fluoroscopy image-guided pedicle screw placement in the thoracolumbar spine. Spinal navigation View project Navigated Transcranial Magnetic Stimulation in Neurosurgery View project. 10.1016/j.spinee.2014.10.003. 10.1016/j.spinee.2014.10.003 25315133

[18] Richter M, Cakir B, Schmidt R (2005). Cervical pedicle screws: Conventional versus computer-assisted placement of cannulated screws. Spine.

[19] Scheufler KM, Franke J, Eckardt A, Dohmen H (2011). Accuracy of image-guided pedicle screw placement using intraoperative computed tomography-based navigation with automated referencing, part I: Cervicothoracic spine. Neurosurgery.

[20] Shin BJ, James AR, Njoku IU, Hartl R (2012). Pedicle screw navigation: A systematic review and meta-analysis of perforation risk for computer-navigated versus freehand insertion. J. Neurosurg. Spine.

[21] Moses Z (2013). Neuronavigation in minimally invasive spine surgery. Neurosurg. Focus.

[22] Mason A (2014). The accuracy of pedicle screw placement using intraoperative image guidance systems. J. Neurosurg. Spine.

[23] Pennington Z (2019). Evaluation of surgeon and patient radiation exposure by imaging technology in patients undergoing thoracolumbar fusion: Systematic review of the literature. Spine J..

[24] Xiao R (2017). Clinical outcomes following spinal fusion using an intraoperative computed tomographic 3D imaging system. J. Neurosurg. Spine.

[25] Hecht N (2016). Accuracy and workflow of navigated spinal instrumentation with the mobile AIRO^®^ CT scanner. Eur. Spine J..

[26] Czerny C (2015). Combining C-arm CT with a new remote operated positioning and guidance system for guidance of minimally invasive spine interventions. J. Neurointerv. Surg..

[27] Oertel MF, Hobart J, Stein M, Schreiber V, Scharbrodt W (2011). Clinical and methodological precision of spinal navigation assisted by 3D intraoperative O-arm radiographic imaging: Technical note. J. Neurosurg. Spine.

[28] Kendlbacher P (2022). Workflow and performance of intraoperative CT, cone-beam CT, and robotic cone-beam CT for spinal navigation in 503 consecutive patients. Neurosurg. Focus.

[29] Deng Y (2015). Effect of surface roughness on osteogenesis in vitro and osseointegration in vivo of carbon fiber-reinforced polyetheretherketone: Nanohydroxyapatite composite. Int. J. Nanomed..

[30] Laufer I (2013). The NOMS framework: Approach to the treatment of spinal metastatic tumors. Oncologist.

[31] Tkatschenko D (2020). Navigated percutaneous versus open pedicle screw implantation using intraoperative CT and robotic cone-beam CT imaging. Eur. Spine J..

[32] Hecht N (2018). Intraoperative computed tomography versus 3D C-Arm imaging for navigated spinal instrumentation. Spine.

[33] Gertzbein SD, Robbins SE (1990). Accuracy of pedicular screw placement in vivo. Spine.

[34] Rampersaud YR, Pik JHT, Salonen D, Farooq S (2005). Clinical accuracy of fluoroscopic computer-assisted pedicle screw fixation: A CT analysis. Spine.

[35] Scarone P (2018). Use of the Airo mobile intraoperative CT system versus the O-arm for transpedicular screw fixation in the thoracic and lumbar spine: A retrospective cohort study of 263 patients. J. Neurosurg. Spine.

[36] Garayoa J, Castro Pablo C (2013). A study on image quality provided by a kilovoltage cone-beam computed tomography. J. Appl. Clin. Med. Phys..

[37] Rampersaud YR, Simon DA, Foley KT (2001). Accuracy requirements for image-guided spinal pedicle screw placement. Spine.

[38] Härtl R (2013). Worldwide survey on the use of navigation in spine surgery. World Neurosurg..

